# False-Positive Malignant Diagnosis of Nodule Mimicking Lesions by Computer-Aided Thyroid Nodule Analysis in Clinical Ultrasonography Practice

**DOI:** 10.3390/diagnostics10060378

**Published:** 2020-06-06

**Authors:** Krisztián Molnár, Endre Kálmán, Zsófia Hári, Omar Giyab, Tamás Gáspár, Károly Rucz, Péter Bogner, Arnold Tóth

**Affiliations:** 1 Department of Diagnostic Imaging, University of Pécs Medical School, Ifjúság út 13, 7624 Pécs, Hungary; bertazsofia013@gmail.com (Z.H.); giyab.omar@pte.hu (O.G.); gaspar.tamas.aok@gmail.com (T.G.); bogner.peter@pte.hu (P.B.); toth.arnold@pte.hu (A.T.); 2 Department of Pathology, University of Pécs Medical School, Szigeti Út 12, 7643 Pécs, Hungary; ke6100@gmail.com; 3 1st Department of Medicine, Division of Endocrinology, University of Pécs Medical School, Ifjúság út 13, 7624 Pécs, Hungary; klinika@somogy.hu

**Keywords:** computer-assisted diagnosis, ultrasonography, thyroid nodule, thyroid cancer, diagnostic errors

## Abstract

This study aims to test computer-aided diagnosis (CAD) for thyroid nodules in clinical ultrasonography (US) practice with a focus towards identifying thyroid entities associated with CAD system misdiagnoses. Two-hundred patients referred to thyroid US were prospectively enrolled. An experienced radiologist evaluated the thyroid nodules and saved axial images for further offline blinded analysis using a commercially available CAD system. To represent clinical practice, not only true nodules, but mimicking lesions were also included. Fine needle aspiration biopsy (FNAB) was performed according to present guidelines. US features and thyroid entities significantly associated with CAD system misdiagnosis were identified along with the diagnostic accuracy of the radiologist and the CAD system. Diagnostic specificity regarding the radiologist was significantly (*p* < 0.05) higher than when compared with the CAD system (88.1% vs. 40.5%) while no significant difference was found in the sensitivity (88.6% vs. 80%). Focal inhomogeneities and true nodules in thyroiditis, nodules with coarse calcification and inspissated colloid cystic nodules were significantly (*p* < 0.05) associated with CAD system misdiagnosis as false-positives. The commercially available CAD system is promising when used to exclude thyroid malignancies, however, it currently may not be able to reduce unnecessary FNABs, mainly due to the false-positive diagnoses of nodule mimicking lesions.

## 1. Introduction

Thyroid nodules are present in 4–68% of the global population [1,2]. Thyroid cancer is the most common malignancy in the endocrine system and is associated with a continuously increasing rate of incidence [3,4].

Fine needle aspiration biopsy (FNAB) is the primary diagnostic tool used to detect malignancies [5,6]. Ultrasonography (US) plays a major role in indicating FNAB [7,8]. US morphological features indicative of malignancy have been extensively studied, and different classification systems have been derived resulting in a more consistent and accurate differentiation among benign and malignant nodules [9,10,11].

Admittedly, there is still relatively high inter- and also an intra-observer discrepancy in nodule evaluation, necessarily resulting in inconsistent and less than desired sensitivity and specificity rates (52–81% and 54–83%, respectively), with both unnecessary biopsies and missed malignancies [12,13,14]. The most likely explanation is that nodule assessment relies highly on experience [15,16,17,18].

With respect to the expectation of achieving a more reliable, objective, and time-saving approach, computer-aided diagnosis (CAD) systems for thyroid nodules regarding US have recently been introduced. These CAD systems have been shown to approximate or even exceed the accuracy among experts in differentiating thyroid nodules in test image sets [19,20,21,22,23,24,25,26,27,28,29]. Only a handful of studies have tested CAD systems in clinical practice, still including only selected cases of true nodules [30,31,32,33]. To date, most CAD systems are not generally available and do not enable real-time use. Despite the slightly varied results, most studies concluded that CAD systems can be best exploited by less experienced users [29,31,32,34,35].

However, the theoretical problem regarding less experienced users applying CAD systems is that these systems were without exception, tested on representative images of true nodules selected by experienced specialists, while in clinical practice, the examiner first needs to differentiate true nodules from mimicking lesions. Moreover, the selection of the most representative plane for analysis itself requires experience [36,37].

To cite an example, the differentiation of a nodule from a pseudonodule or focal parenchymal inhomogeneity may be challenging in chronic or subacute thyroiditis [37,38,39,40,41]. This, in turn, is a very common and important clinical problem, since autoimmune thyroiditis has a very high (up to 20%) prevalence and is also widely believed to pose a higher risk regarding thyroid malignancies [42,43].

The aim of this study is to test the accuracy of CAD in true clinical thyroid US practice by including not only true nodules pre-selected by experts as in earlier studies, but mimicking lesions as well. The study also aims to identify factors and thyroid entities related to systematic CAD errors.

## 2. Materials and Methods

### 2.1. Subjects

**Figure 1:**
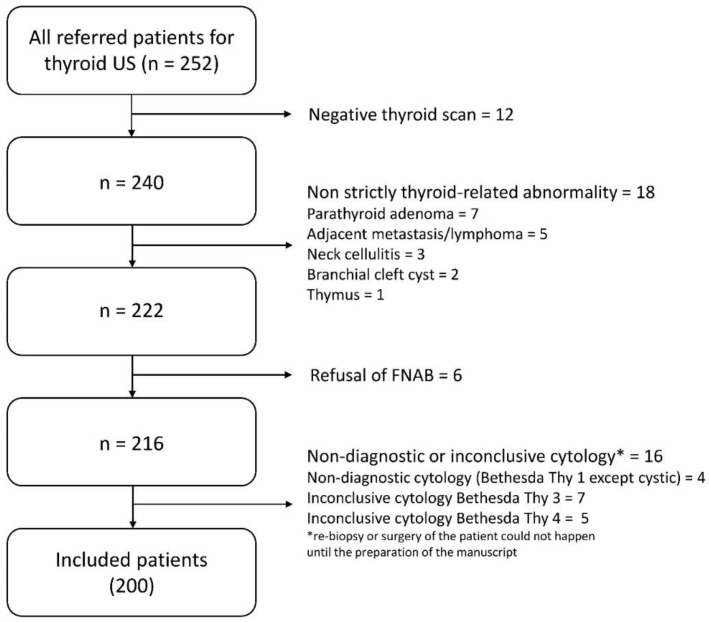
Flow chart of the study population selection. US = Ultrasonography. FNAB = Fine Needle Aspiration Biopsy.

In this prospective study, 200 consecutive patients were included (167 women, 33 men, average age = 53.5 years, range = 12–88 years) who were referred to the Department of Radiology of the University of Pécs, for thyroid US from 2019 February to 2019 June and deemed suitable regarding the study. The exclusion criteria included negative thyroid US, diagnoses of anatomical variants, and diagnoses of non-strictly thyroid related pathologies (e.g., parathyroid adenoma, adjacent abscess, adjacent malignancy of other origin, etc.), since the CAD system used in the present study (see CAD analysis) was developed to analyze only thyroid lesions. Further exclusion criteria included refusal of FNAB, and non-diagnostic or inconclusive FNAB (i.e., Thy 1, Thy 3, and Thy 4 according to the Bethesda system for reporting thyroid cytopathology [44]) without the possibility of re-biopsy or surgery until the preparation of the manuscript. The reason for excluding these cases was to be able to clearly dichotomize nodules as “benign” or “requiring surgery/malignant” (see diagnosis definitions) as the study target outcome. Figure 1 depicts the flow chart of the study population selection.

All patients who underwent FNAB signed the general institutional informed consent regarding the advantages and risks of FNAB and the possible use of anonymized data for research purposes. The institutional review board approved the use of anonymized patient data in support of this study and waived the need for additional informed consent, since the study did not burden patients with other additional procedures than necessary based on the present clinical recommendations [8,11,45] (Code: No. 7751-PTE 2019. date: 10 January 2019).

### 2.2. Ultrasonography (US) Examination and Fine Needle Aspiration Biopsy (FNAB), Diagnosis Definitions

All US examinations were performed using a high-end, real-time US system (RS85 A; Samsung Medison Co. Ltd., Seoul, Korea) and a 3–12 MHz linear probe at a fixed frequency of 10 MHz. Patients were examined by K.M., a radiologist specializing in head and neck radiology with over ten years of experience regarding diagnostic and interventional thyroid US.

Standard thyroid US examination was performed with all patients in a supine position and their neck in hyperextension. Neck lymph node regions, major vessels of the neck, and major salivary glands were also scanned; however, their findings were not included in the study.

If a thyroid nodule was present, the radiologist evaluated its US morphological features presented in Table 1. Based on these features, a K-TIRADS score (Korean Thyroid Imaging Reporting and Data System) was assigned (2 = benign, 3 = low suspicion, 4 = intermediate suspicion, and 5 = high suspicion) [8]. The reason for applying this score system was that since it is integrated in the applied CAD (see CAD analysis), direct comparisons could be performed. A nodule was regarded possibly benign with a K-TIRADS score of 2 or 3, while possibly malignant when associated with a score of 4 or 5.

Additional nodule features were asserted, such as “coarse calcification,” if macrocalcification was present with the largest diameter exceeding 50% of the nodule’s largest diameter, and “inspissated colloid cystic nodule” for well-circumscribed, completely avascular, not entirely anechoic nodules with colloid particles producing a comet tail artifact, which were completely evacuated during aspiration.

If a patient was afflicted with more than one nodule, one showing the highest risk of malignancy, or in the case of more nodules with the same malignancy risk, the largest nodule was included in the study. An axial plane B-mode image of all included nodules at their largest diameters was saved for further CAD analysis.

FNAB indication was based on the present international guidelines [8,11,45] and performed by K.M. In case of discrepancy, the guideline indicating FNAB was applied. US-guided FNAB was performed using the parallel needle to probe technique with a 22 G needle using 10 mL syringe and Cameco biopsy gun; the nodules were panned across to sample their possibly largest portion. The aspirated material was rapidly expressed onto two glass slides, and two smears were created using the one-step smear method. One slide was fixed in 95% ethanol for H&E staining, and one was air-dried for May–Grünwald Giemsa staining. The rest of the obtained material was rinsed in formaldehyde solution for processing as a cell block. Aspiration was repeated if the material macroscopically appeared to be scanty or bloody. The cytological specimen was submitted to the cytopathology laboratory along with all relevant clinical and US information. The cytological analysis was performed by a cytopathologist (E.K.) with over 20 years of experience in cytopathology. Results were classified according to the Bethesda system regarding reporting thyroid cytopathology [44].

Patients with thyroiditis were included in the study. Thyroiditis criteria in the present study included clinically [46,47,48] and radiologically [37,38,39,40,41] established thyroiditis, or thyroiditis substantiated by FNAB.

In patients suffering from thyroiditis, the following categories were specified: (a) focal inhomogeneity, proven not to be a nodule by biopsy or if it was completely unchanged compared with previous examinations within at least a 2-year timespan and was consistently regarded to be thyroiditis related focal inhomogeneity by the examiner; (b) pseudonodule substantiated through biopsy or was completely unchanged compared with previous examinations of at least a 2-year timespan and was consistently regarded as a pseudonodule by the examiner; (c) true nodule in addition to thyroiditis, which was managed in the same way as nodules without thyroiditis. In reference to focal in homogeneities and pseudonodules, the examiner assigned a K-TIRADS score of 1 (no nodule) for further statistical analyses.

An axial plane B-mode image representing these entities at their largest diameters were also saved for further CAD analysis to assess the accuracy of nodule detection in thyroiditis.

Nodules were regarded malignant or requiring surgery (referred to as “malignant/surgery” in further texts) if the cytological result was suspicious regarding malignancy (Thy 5), or malignant (Thy 6), and/or malignancy was evident in the surgical specimen. A benign nodule was diagnosed when any of the following criteria were met: (i) confirmation of benign status in a surgical specimen; (ii) benign or cystic cytology of an FNAB (Thy 1c or Thy 2); (iii) benign traits including spongiform or partially cystic nodules with comet tail artifacts, or pure cysts evident on US; (iv) low suspicion (K-TIRADS 3) nodules under 15 mm diameter, which were completely unchanged compared with previous examinations of at least a 2-year timespan, and no clinical poor prognostic factors were present, and therefore, FNAB was not indicated.

### 2.3. Computer-Aided Diagnosis (CAD) Analysis

S-Detect 2 for thyroid (Samsung Medison Co., Ltd.), which is a commercially available CAD tool integrated into the real-time US system (Samsung RS85 A) designed to detect and classify thyroid lesions was used in the study. S-Detect 2 for thyroid is based on convolutional neural network-based deep learning techniques. S-Detect evaluations were performed offline, so the primary ultrasonography examiner (K.M.) was blinded to the CAD outcomes. The CAD evaluation was performed by consensus by O.G. and A.T., a radiologist with over 5 years of experience in thyroid imaging and a resident with 32 months of supervised experience in thyroid imaging, respectively, blinded both to the findings of the primary radiological evaluation and the cytopathological results. The analysis was run on the axial plane images of the nodules, focal inhomogeneities related to thyroiditis, pseudonodules in thyroiditis, and true nodules besides thyroiditis stored and marked by the primary examiner (K.M.). The CAD data were obtained by manually setting a rectangular region of interest around the lesion. The CAD system suggested four different possible margins for the detected nodule; however, the default one was always used. The software automatically evaluated the US features of the nodule presented in Table 1. The system is able to incorporate nodule elasticity and vascularity upon user selection, but these features were omitted in this study. This system can be set up to provide a simple output as “possibly benign” or “possibly malignant” or to provide a K-TIRADS score of the lesions. The latter option was used to achieve a more detailed evaluation. A lesion was regarded possibly CAD benign if the provided K-TIRADS score was 2 or 3, while a nodule with provided K-TIRADS score of 4 to 5 was regarded as possibly CAD malignant.

### 2.4. Statistical Analysis

Regarding statistical analysis, data were analyzed using MedCalc Statistical Software, version 18.11.3 (MedCalc Software bvba, Ostend, Belgium, https://www.medcalc.org; 2019) [49].

First, we aimed to identify US features and entities associated with CAD system misdiagnosis; therefore, we selected cases in which the radiologist’s diagnosis was correct and created two subgroups: one in which the CAD was correct and another in which the CAD was incorrect. Between these groups (CAD correct vs. CAD incorrect), the rates of entities such as focal inhomogeneity related to thyroiditis and pseudonodule related to thyroiditis (as defined earlier) were statistically compared using the comparison of the two rates tool [50]. Next, only cases of true nodules were kept in the CAD correct and CAD incorrect groups (focal inhomogeneity related to thyroiditis and pseudonodule related to thyroiditis cases were excluded), and the rates of nodule US features assured by the radiologist (coarse calcification, macrocalcification without coarse calcification, inspissated colloid cystic nodule, true nodule related to thyroiditis, composition, echogenicity, orientation, margin, spongiform state, shape, and microcalcification) were statistically compared including the comparison of the two rate tools [50].

Secondly, to assess the effect of these entities and US features related to CAD system misdiagnosis regarding the overall diagnostic performance, the receiver operating characteristic (ROC) curves with K-TIRADS scores provided by the examiner or CAD as variables and benign or malignant/surgery diagnosis as a classification variable in the following groups were compared using the comparison of independent ROC curves with the methodology by DeLong et al. [51,52,53,54,55]: (a) total cohort, human rating, (b) total cohort, CAD rating, (c) a subgroup derived from the total cohort by excluding all cases in which the entities or US features identified to be significantly associated with CAD system misdiagnosis were present (=“screened subgroup”), CAD rating, and (d) the same screened subgroup, human rating.

Third, among these four groups, the sensitivity, specificity, and accuracy were compared using the McNemar test in reference to dependent sample comparisons and Pearson’s Chi-squared test for independent samples.

All these steps regarding statistical analysis were additionally run in the group including only those patients who had an FNAB (FNAB-only group).

As an ancillary step, the number of cases in which the radiologist’s diagnosis was incorrect but the CAD system diagnosis was correct was calculated.

Tests resulting with a *p*-value of <0.05 were considered statistically significant.

## 3. Results

Table 2 presents the occurrence of cases and diagnoses.

### 3.1. US Features or Entities Associated with CAD System Misdiagnosis Including Mimicking Lesions

**Figure 2:**
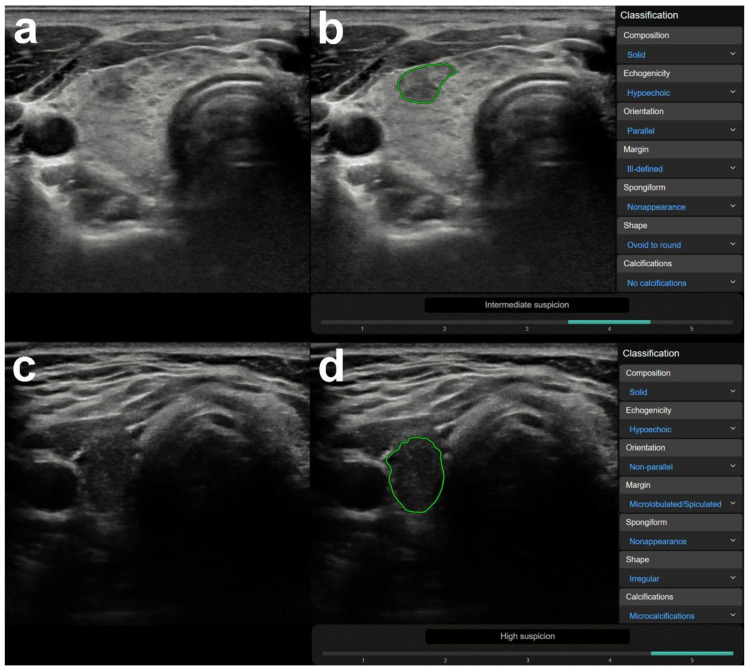
Representative images of computer-aided diagnosis (CAD) system false-positive misdiagnoses of focal inhomogeneities related to thyroiditis. (**a**) (B-mode thyroid US, axial) A 26-year-old female patient with clinically obvious Hashimoto thyroiditis. Surrounded by diffuse hypoechogenic inhomogeneity of the thyroid gland, a more circumscribed inhomogeneity is present in the ventral part of the right lobe. This appearance went unchanged for over three years of follow up in our department, rated as no nodule (K-TIRADS 1) by the radiologist. The region of interest for CAD analysis was placed over the circumscribed inhomogeneity. (**b**) (CAD output image) The CAD system interpreted the lesion as a nodule and rated possibly malignancy and a K-TIRADS 4 score. (**c**) (B-mode thyroid US, axial) A 31-year-old female patient also with clinically obvious Hashimoto thyroiditis. The thyroid appears diffusely hypoechogenic and a thyroid septum is visible in the right lobe causing the posterior part of the lobe mimicking a nodule. This appearance was unchanged for over 4 years of follow up in our department, rated as no nodule (K-TIRADS 1) by the radiologist. The region of interest for CAD analysis was placed over the posterior part of the right lobe encased by the septum. (**d**) (CAD output image) The CAD system interpreted the lesion as a nodule and rated possibly malignancy and a K-TIRADS 5 score.

In 176 out of the 200 cases, the radiologist made a correct diagnosis. Out of these 176 cases, the CAD was correct in 83 and incorrect in 93 cases. Focal inhomogeneities related to thyroiditis were in a significantly higher rate present in the CAD incorrect group; the CAD system identified these lesions as nodules and assigned them a median K-TIRADS score of 5 (see Table 3). Figure 2 shows representative cases of focal inhomogeneity related to CAD system misdiagnosis.

### 3.2. US Features or Entities Associated with CAD System Misdiagnosis Excluding Mimicking Lesions

**Figure 3:**
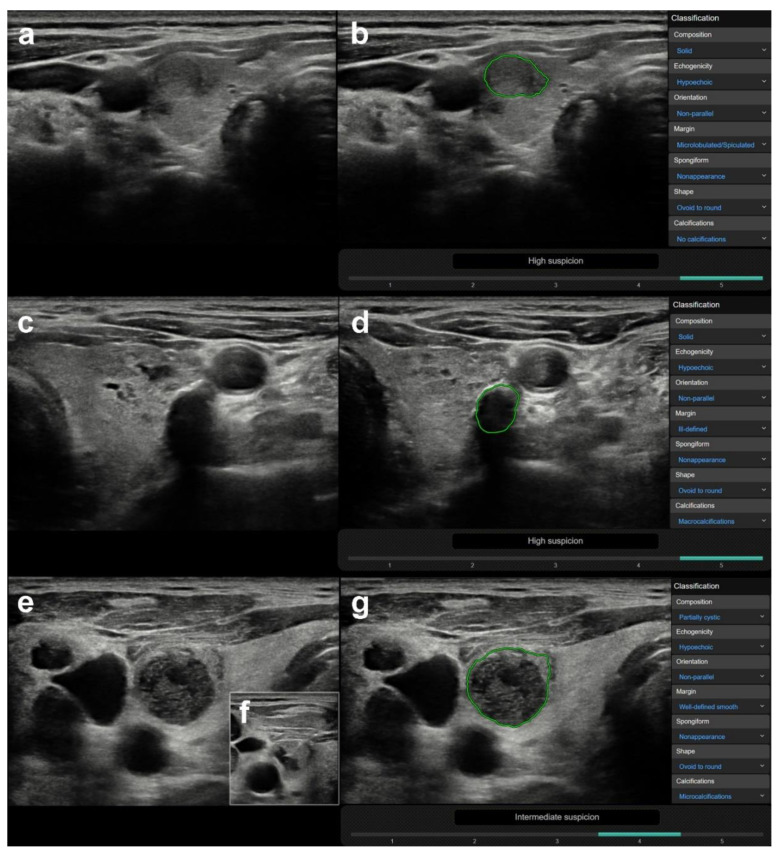
Representative images of CAD system false-positive misdiagnoses of nodules ((**a**,**b**) nodule besides thyroiditis, (**c**,**d**) nodule with coarse calcification, (**e**–**g**) inspissated colloid cystic nodule). (**a**) (B-mode thyroid US, axial) A 64-year-old female patient with clinically known Hashimoto thyroiditis. In addition to focal inhomogeneities due to thyroiditis, a true nodule can be depicted in the right lobe, regarded benign and K-TIRADS 3 by the radiologist. FNAB was performed and yielded a benign result (Thy 2). (**b**) (CAD output image) The CAD system provided a high suspicion for malignancy (K-TIRADS 5) diagnosis. (**c**) (B-mode thyroid US, axial) A 63-year-old male patient with confluent well circumscribed isoechoic, partially cystic nodules in the left thyroid lobe with a coarse macrocalcification, scored K-TIRADS 3 nodule by the radiologist. FNAB provided benign diagnosis (Thy 2). (**d**) (CAD output image) The CAD system yielded a result of high suspicion for malignancy (K-TIRADS 5). (**e**) (B-mode thyroid US, axial) A 71-year-old male patient presenting with several clinically and radiologically pathological lymph nodes in right cervical lymph node regions and a nodule in the right thyroid lobe, which was well circumscribed, completely avascular, contained echogenic foci with comet tail artefacts, and was hypoechogenic. The radiologist diagnosed an inspissated colloid cystic nodule (K-TIRADS 2), yet performed FNAB due to the presence of pathological lymph nodes. (**f**) (B-mode thyroid US, axial, insert) During FNAB, the fluid content of the nodule was completely removed. The pathological lymph nodes were proved to be squamous cell carcinoma metastases, while the thyroid nodule was diagnosed benign (Thy 1c) by cytology. (**g**) (CAD output image) The CAD system rated the nodule to be possibly malignant, with an intermediate suspicion for malignancy (K-TIRADS 4).

**Figure 4:**
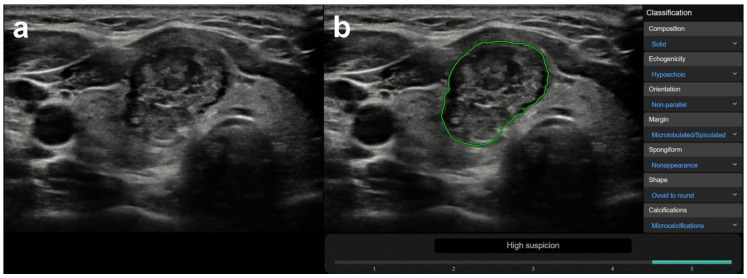
Representative image of a malignant nodule (**a**), correctly diagnosed by the CAD system (**b**). (**a**) (B-mode thyroid US, axial) This 26-year-old female patient had a nodule in the right thyroid lobe characterized as solid, hypoechoic, non-parallel, ill-defined, and irregularly shaped with microcalcifications by the radiologist. (**b**) (CAD output image) Although the CAD system did not agree in all US classification features, the outcome of high suspicion regarding malignancy (K-TIRADS 5) concurred with the radiologist’s diagnosis. Cytology and histology confirmed the presence of papillary thyroid cancer.

CAD was correct in 78 case and incorrect in 64 cases, regarding true nodules within the group in which a correct diagnosis was made by the radiologist (*n* = 142). True nodules related to thyroiditis, coarse macrocalcifications, and inspissated colloid cystic nodules were in a significantly higher rate present in the CAD incorrect group vs. the CAD correct group, with median CAD system K-TIRADS scores of 4, 5, and 4, respectively, while only one truly malignant case was present within these groups (Table 3). Figure 3 shows representative cases of these US features related to CAD system misdiagnosis. Non-parallel orientation, ill-defined margin, and irregular shape were in a significantly higher rate present in the CAD correct group and they were all malignant/surgery cases (Table 3). A representative example is shown in Figure 4.

Results of the same tests run in the FNAB only group (*n* = 121) are presented in Supplementary Materials Table S1.

Out of the 24 cases where the radiologist’s diagnosis was incorrect, the CAD was correct in four cases. In all of them the radiologist gave a TIRADS score of 4, while the CAD gave a TIRADS score of 3, and these cases were proven to be benign by cytology.

### 3.3. Comparison of Human and CAD System Diagnostic Performance in the Total and in the Screened Subgroup

Regarding all cases, human specificity (88.1%) and accuracy (88%) in detecting malignancies were significantly higher than when compared with those of the CAD (40.5% and 43.5%, respectively). There was no significant difference in sensitivity (human sensitivity = 88.6%, CAD sensitivity = 80%). ROC curve comparison showed a significant difference in areas under the curves (AUROCs), which were 0.937 for the human detections and 0.656 for the CAD detections.

The exclusion of cases of US features and entities identified to be related to CAD system misdiagnosis (cases of focal inhomogeneity in thyroiditis, true nodule in thyroiditis, coarse macrocalcification, and inspissated colloid cystic nodule) from the study population resulted in a “screened” subgroup including 148 cases. In this group, a significant improvement in the specificity of CAD compared to its specificity achieved in all cases could be detected; specificity in the screened subgroup increased to 55.9%. However, no significant change was observed regarding sensitivity, accuracy, and AUROC.

The comparison of human and CAD diagnostic performance in the screened subgroup showed similar results as in the group of all patients since the difference in specificity, accuracy, and AUROC remained significant, while sensitivity was not significantly different.

Neither diagnostic parameters showed a significant difference among the total and the screened subgroup human detections.

Table 4 shows details of diagnostic parameters of human and CAD detections in the total population and in the screened subgroup, including their comparisons.

**Figure 5:**
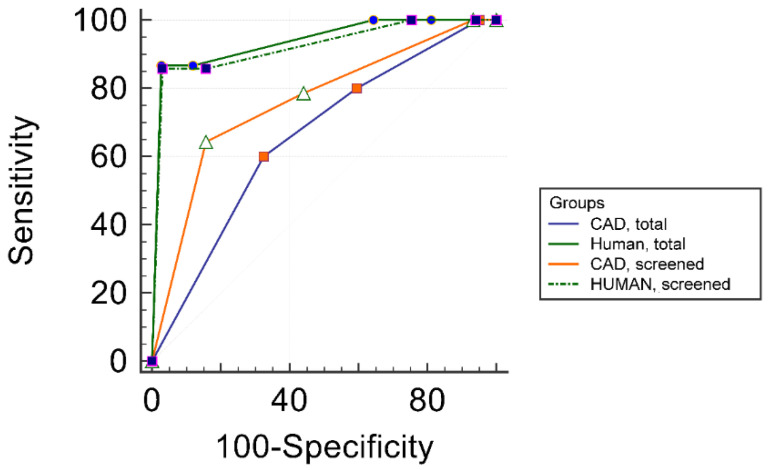
Comparison of ROC curves of the radiologist and CAD system in the total cohort and the screened subgroup (in which cases of thyroid entities and nodule features identified to be associated with CAD system misdiagnosis were excluded) for detecting malignancy and nodules requiring surgery.

Figure 5 shows ROC curves yielded by human and CAD in the total population and in the screened subgroup.

Results of the same tests conducted in the FNAB-only group (*n* = 121) are presented in Supplementary Materials Table S2.

## 4. Discussion

To the best of our knowledge, no previous study has considered the importance of nodule mimicking lesions when applying CAD systems for thyroid. CAD systems were trained on true nodules and often shown to even outperform humans. In our opinion, such results may be very misleading regarding the actual feasibility of CAD systems, since they do not represent clinical practice, in which thyroid lesion differentiation (nodule vs. mimicking lesion) is of utmost importance. Such differentiation might be challenging, especially for less experienced users—the ones most probably willing to apply CAD. To test the significance of this problem, the present study focused not only on true nodules but mimicking lesions as well.

The overall diagnostic performance (AUROC and accuracy) regarding the experienced radiologist was comparable to previous studies [30,31,33], and was significantly higher when compared with the CAD system. The most substantial difference was found in specificity and positive predictive value, which were, respectively, roughly two and four times higher for the radiologist’s detections. However, there was no significant difference in sensitivities, and negative predictive values were also very close. In the study by Kim et al. [32], who applied the same commercial CAD system S-Detect 2, CAD sensitivity (81.4%) and negative predictive value (84.9%) vs. radiologist (sensitivity = 84.9%, negative predictive value = 90.7%) were also similar, and CAD specificity (68.2%) was also significantly lower than that of the radiologist (96.2%). However, in our study, CAD specificity was even lower (40.5%). This is most likely due to the fact that in our study, not only cases of true nodules pre-selected by an expert were included, but also cases posing differential diagnostic problem for nodules such as focal inhomogeneities as well. Another important difference regarding the populations of the two studies is that Kim et al. included patients who were prior to scheduled surgery, and almost half of the analyzed nodules were malignant, while in the present study most patients underwent US for the first time or returned for a check-up of a benign thyroid entity, which resulted in a lower proportion of malignancies.

Thyroiditis related focal inhomogeneity appeared to be a differential diagnostic entity related to systematic CAD system misdiagnosis, i.e., false-positive detection, since the CAD system appreciated them as a nodule and almost always assigned them a K-TIRADS score of 5 due to “ill-defined borders” and “hypoechogenicity”. In clinical practice, especially with less experienced users, such false-positive misdiagnoses may lead to high rates of unnecessary FNAB indications, keeping the high incidence of chronic thyroiditis in mind [42,43].

When considering true nodules, CAD system misdiagnosis was most strikingly related to nodules associated with coarse macrocalcifications. All of them were diagnosed false-positively as possibly malignant and were mostly assigned a K-TIRADS score of 5. We assume this is due to the acoustic shadow being assessed as solid hypoechogenicity with ill-defined borders. Inspissated colloid cystic nodules, proved by the aspiration of their fluid content and cytology, were again diagnosed as possibly malignant and were assigned a K-TIRADS score of 4 or 5 nodules by the CAD, mostly diagnosed with solid hypoechoic composition and microcalcification instead of colloid particles. The likelihood of CAD being inaccurate while evaluating microcalcification was also presented by Kim et al. [32].

US features such as ill-defined contour, non-parallel orientation, and irregular shape were, in turn, significantly associated with correct diagnosis by the CAD system. This is aligned with the finding regarding high CAD sensitivity, since all of these features are known to be associated with the risk of malignancy [8] and seem to be accurately picked up by the CAD.

In contrast with our results, several studies of non-commercial, yet offline applicable algorithms presented artificial diagnostic performance being as good or even better than radiologists’ performance [19,20,29,34,56]. These studies included a very high (approximately 30–50%) rate of malignancies compared to “real life” thyroid malignancy incidence and malignant nodule rate [3,4,6]. Furthermore, in all these studies, the validation sets included only true nodules pre-selected by experts. Such nodule pre-selection and the acquisition of the most representative slice performed by humans is obviously a diagnostic procedure possibly significant in helping CAD systems to achieve their promising diagnostic performance results. This is underscored by our result in which the exclusion of cases posing thyroid nodule differential diagnosis (see results related to the exclusion of the “screened subgroup”) significantly improves CAD outcomes. Moreover, Jeong et al. [36] showed how CAD outcomes are significantly operator dependent, even if operators run the analysis on exactly the same images of pre-selected nodules; however, they may differently position the nodule region of interest and select nodule contours.

In this study, the radiologist had the possibility to consider clinical data and scan the entire lesions for lesion differentiation and scoring. This might have constituted an advantage regarding diagnostic performance versus the CAD system, which could not rely on clinical data, and analyzed the lesions based on single slice images. However, the aim of this study was to make a comparison under true clinical circumstances and to find CAD limitations. To overcome such possible CAD shortcomings, the authors speculate that future CAD systems should have the option of including clinical data and the option of analyzing 3D inputs. Some attempts towards 3D nodule analysis have been already done [57,58].

It is important to note that there are certain differences among the different TIRADS systems (such as ACR and EU TIRADS) compared to the presently applied K-TIRADS; for a comparison of these systems, see the review by Chiara et al. [59].

This study has certain limitations. First, not all patients underwent FNAB. In these cases, however, the chance of malignancy was firmly ruled out according to strict criteria. The authors alleged including these cases to be relevant in effectively evaluating CAD performance in routine clinical practice and not only on human-selected nodules requiring FNAB. Still, the inclusion of non-FNAB cases carries the possibility of bias since the examination was performed by a single expert. Therefore, we ran all statistics in the FNAB-only group as well (see Supplementary Materials), which did not affect the main messages of the study. Second, in this study not only histologically confirmed malignancies or cytological Thy 6 cases were included as positives (malignant/surgery), but cytological Thy 5 cases as well, since we believe that the aim of thyroid US is primarily to detect nodules that require surgery and not substituting FNAB by attempting to provide final diagnosis. The number of Thy 5 cases without final histological diagnosis was, however, low (4 cases out of 200, 2%), and in all except of one of these cases, the CAD outcome (possibly malignant) was correct and was in agreement with the human outcome; therefore, including these cases did not affect the main findings of the present study related to false positive CAD results. Third, no correction for multiple comparisons was performed when identifying US entities and features possibly related to CAD system misdiagnosis. Nevertheless, the fact that all cases in these groups (except one truly malignant nodule related to thyroiditis) were false positive, and the exclusion of these cases significantly affected diagnostic specificity, is reassuring regarding the validity of this finding. Fourth, only a single CAD system was tested in this study; however, the results regarding misdiagnosis of nodule mimicking lesions most probably apply to all other thyroid US CAD systems, since to date none of them were reported to consider mimicking lesions. Fifth, it was not possible to analyze the causes of false-negative CAD detections because of the very low number of these cases (*n* = 3).

## 5. Conclusions

In a routine clinical thyroid US population, the commercially available CAD seems to be applicable for screening patients with the aim of excluding thyroid malignancies. However, certain nodule types, and especially mimicking lesions, resulted in systematic false-positive malignant diagnoses for this CAD system. Therefore, this system (and probably any system trained on true nodules only) may not be entirely effective in reducing unnecessary FNABs, especially when used by inexperienced users for whom the diagnosis of the above-mentioned entities may also prove daunting.

Future CAD systems regarding thyroid may be most useful in clinical practice if mimicking lesions were added to their training sets.

## Figures and Tables

**Table 1:** Nodule ultrasonography (US) features assessed by the radiologist and the computer-aided diagnosis (CAD) system.

US Feature	Outcome
Composition	Solid
	Partially cystic
	Cystic
Echogenicity	Hyper/isoechogenic
	Hypoechogenic
Orientation	Parallel
	Non-parallel
Margin	Well-defined
	Microlobulated
	Ill-defined
Spongiform	Appearance
	Non-appearance
Shape	Oval-to-round
	Irregular
Calcification	No calcification
	Macrocalcification
	Microcalcification ^1^

^1^ with or without macrocalcification.

**Table 2:** Occurrence of thyroid cases and diagnoses.

Case/Diagnosis/Feature		Number
Malignant-surgery diagnosis/all		15 ^a^/200
Radiologist possible malignancies (/correct)		35 (/13)
CAD system possible malignancies (/correct)		122 (/12)
Radiologist missed malignancies		2
CAD system missed malignancies		3
Thyroiditis ^b^ cases	all	43
	with true nodules	9
	with pseudonodules	8
	with focal inhomogeneities	26
Nodule features of interest	coarse macrocalcification	12
	non-coarse macrocalcification	10
	inspissated colloid	5
FNAB	all	121
	in focal inhomogeneity	14
	in nodule with coarse macrocalcification	7
	in nodule with non-coarse macrocalcification	4
	in inspissated colloid cystic nodule	4
	in true nodule related to thyroiditis	5
Radiologist K-TIRADS scores 1/2/3/4/5		34/31/100/17/18
CAD system K-TIRADS scores 1/2/3/4/5		0/9/69/53/69
Lesion size (largest diameter, mm)	min	8
	max	42
	average	14

^a^ 11 histologically malignant cases (10 papillary cancer, 1 follicular cancer) and 4 cases of a result of Bethesda system Thy 5; ^b^ 38 Hashimoto thyroiditis, 2 subacute thyroiditis, and 4 Graves’ disease cases.

**Table 3:** Relationship between thyroid entities, US nodule characteristics, and CAD accuracy.

Thyroid Entities *		Rate		*p* ^2^	CAD TIRADS ^3^ Rates					Malignancies/Diagnosed ^4^
		CAD ^1^ Correct Group	CAD Incorrect Group		1	2	3	4	5	
Mimicking lesions	**Focal inhomogeneity (thyroiditis)**	**0**	**26**	**<0.0001**	**0**	**0**	**0**	**2**	**24**	**0/0**
	Pseudonodule in thyroiditis	5	3	0.48	0	0	5	0	3	0/0
True nodules	**True nodule in thyroiditis**	**1**	**8**	**0.019**	**0**	**0**	**0**	**8**	**1**	**1/1**
	Macrocalcification non-coarse	4	5	0.527	0	0	3	3	3	0/0
	**Coarse macrocalcification**	**0**	**12**	**0.0001**	**0**	**0**	**0**	**1**	**11**	**0/0**
	**Inspissated colloid cystic nodule**	**0**	**5**	**0.014**	**0**	**0**	**0**	**3**	**2**	**0/0**
US features *										
Composition	Solid	30	30	0.442	0	0	18	16	26	12/12
	Partially cystic	34	29	0.878	0	2	33	22	6	1/0
	Cystic	14	5	0.1	0	7	7	4	1	0/0
Echogenicity	Hyper/isoechoic	58	54	0.5	0	6	53	37	16	1/0
	Hypoechoic	20	10	0.2	0	3	5	5	17	12/12
Orientation	Parallel	68	63	0.487	0	9	57	41	24	4/3
	*Non-parallel*	*10*	*1*	*0.017*	*0*	*0*	*1*	*1*	*9*	*10/10*
Margin	Well-defined	65	58	0.642	0	9	56	37	21	2/1
	Microlobulated	5	5	0.754	0	0	2	4	4	3/3
	*Ill-defined*	*8*	*1*	*0.04*	*0*	*0*	*0*	*1*	*8*	*8/8*
Spongiform	Appearance	11	8	0.8	0	2	9	7	1	0/0
	Non-appearance	67	56	0.919	0	7	49	35	32	13/12
Shape	Ovoid to round	71	64	0.585	0	9	58	40	28	6/5
	*Irregular*	*7*	*0*	*0.017*	*0*	*0*	*0*	*2*	*5*	*7/7*
Microcalcification		5	1	0.162	0	0	1	1	4	6/5

^1^ Computer aided diagnosis; ^2^ *p* value for comparison of rates; *^3^* K-TIRADS score; ^4^ malignant cases and cases requiring surgery/correctly diagnosed malignancies and cases requiring surgery by CAD per subgroups; lines with bold letters indicate entities and US nodule characteristics significantly associated with CAD misdiagnosis; lines with italic letters indicate US nodule characteristics significantly associated with correct CAD system diagnosis; * based on the radiologist’s evaluation, including only those cases (*n* = 176), in which the radiologist’s diagnosis was proven correct by either cytology (if performed based on present recommendations) or clinical data (see methods diagnosis definitions). For US feature analyses, only cases of true nodules were included (*n* = 142).

**Table 4:** Diagnostic parameters of human and CAD ^1^ detections in the total and screened ^2^ subgroup for malignancies ^3^.

Diagnostic Parameter	Human, All	CAD, All	CAD, Screened	Human, Screened	*p* Values of Comparisons ^4^			
					Human vs. CAD All	Human vs. CAD Screened	CAD all vs. Screened	Human All vs. Screened
Sensitivity	88.67%	80%	78.57%	85.71%	1	1	0.92	0.94
Specificity	88.11%	40.54%	55.97%	84.33%	<0.0001	<0.0001	0.007	0.33
Accuracy	88%	43.5%	58.1%	84.46%	<0.0001	<0.0001	0.12	0.8
PPV ^5^	37.14%	9.84%	15.71%	36.36%				
NPV ^6^	98.79%	96.15%	96.15%	98.26%				
ROC AUC ^7^	0.937	0.656	0.76	0.922	0.0002	0.049	0.289	0.782

^1^ Computer aided diagnosis; ^2^ subgroup in which cases of thyroid entities and US characteristics identified to be associated with CAD misdiagnosis are excluded (see Table 1); ^3^ malignant cases or cases requiring surgery; ^4^ McNemar test for paired comparisons, Pearson’s chi squared test for unpaired comparisons; ^5^ positive predictive value; ^6^ negative predictive value; ^7^ receiver operator characteristic area under the curve.

## Supplementary Materials

The following are available online at https://www.mdpi.com/2075-4418/10/6/378/s1, Table S1: Relationship between thyroid entities, US nodule characteristics, and CAD accuracy in the FNAB-only group. Table S2: Diagnostic parameters of human and CAD detections in the total and screened subgroup for malignancies in the FNAB-only group.
