# Supplementary material for: False-Positive Malignant Diagnosis of Nodule Mimicking Lesions by Computer-Aided Thyroid Nodule Analysis in Clinical Ultrasonography Practice
Source: Diagnostics (Basel). 2020 Jun 6;10(6):378. doi: 10.3390/diagnostics10060378 (PMC7345888; doi:10.3390/diagnostics10060378)
Supplement: Supplementary file 1 [file diagnostics-10-00378-s001.pdf]

Supplementary Table 1. Relationship between thyroid entities, US nodule characteristics and CAD accuracy in the FNAB-only group

Supplementary table 1. Relationship between thyroid entities, US nodule characteristics and CAD accuracy in the FNAB-only group

Thyroid entities*		Rate: CAD[1] Correct group	Rate: CAD Incorrect group	p[2]	CAD TIRADS[3] rates: 1	2	3	4	5	Malignancies/ diagnosed[4]
Mimicking lesions	**Focal inhomogeneity (thyroiditis)**	**0**	**7**	**0.0032**	**0**	**0**	**0**	**0**	**7**	**0/0**
	Pseudonodule in thyroiditis	0	1	0.267	0	0	0	1	0	0/0
True nodules	True nodule in thyroiditis	1	1	0.879	0	0	0	2	0	1/1
	Macrocalcification non-coarse	3	4	0.553	0	0	2	2	3	0/0
	**Coarse macrocalcification**	**0**	**7**	**0.0004**	**0**	**0**	**0**	**0**	**7**	**0/0**
	**Inspissated colloid cystic nodule**	**0**	**4**	**0.03**	**0**	**0**	**0**	**3**	**1**	**0/0**
US features*										
Composition	Solid	24	17	0.729	0	0	12	10	19	12/12
	Partially cystic	23	13	0.738	0	1	23	8	4	1/0
	Cystic	5	3	0.934	0	3	2	3	0	0/0
Echogenicity	Hyper/isoechoic	37	28	0.482	0	3	35	17	10	1/0
	hypoechoic	15	5	0.205	0	1	3	4	13	12/12
Orientation	Parallel	43	32	0.495	0	4	37	20	14	4/3
	Non-parallel	*10*	*1*	*0.043*	*0*	*0*	*1*	*1*	*9*	*10/10*
Margin	Well-defined	40	30	0.489	0	4	36	19	11	2/1
	Microlobulated	4	3	0.826	0	0	1	2	4	3/3
	Ill-defined	*8*	*0*	*0.023*	*0*	*0*	*0*	*0*	*8*	*8/8*
Spongiform	Appearance	6	1	0.183	0	1	4	0	2	0/0
	Non-appearance	46	32	0.689	0	3	33	21	21	13/12
Shape	Ovoid to round	45	33	0.528	0	4	37	19	18	6/5
	Irregular	*7*	*0*	*0.035*	*0*	*0*	*0*	*2*	*5*	*7/7*
Microcalcification		5	1	0.265	0	0	1	1	4	6/5

[1]computer aided diagnosis [2]p value for comparison of rates [3]K-TIRADS score [4]malignant cases and cases requiring surgery/correctly diagnosed malignancies and cases requiring surgery by CAD per subgroups
Lines with bold letters indicate entities and US nodule characteristics significantly associated with CAD misdiagnosis.
Lines with italic letters indicate US nodule characteristics significantly associated with correct CAD system diagnosis.
*Based on the radiologist's evaluation, including only those cases, in which the radiologist's diagnosis was proven correct by cytology.

Supplementary Table 2. Diagnostic parameters of human and CAD detections in the total and screened subgroup for malignancies in the FNAB-only group

Supplementary table 2. Diagnostic parameters of human and CAD[1] detections in the total and screened[2] cohort for malignancies[3] in the FNAB-only group

					P values of comparisons[4]			
	Human, all	CAD, all	CAD, screened	Human, screened	Human vs CAD all	Human vs CAD screened	CAD all vs screened	Human all vs screened
Sensitivity	86.67%	80%	78.57%	85.71%	1	1	0.92	0.94
Specificity	80.20%	43.56%	54.32%	76.54%	**<0.0001**	**0.0005**	0.15	0.55
Accuracy	81.03%	48.27%	57.89%	77.89%	**<0.0001**	**0.0003**	0.19	0.47
PPV[5]	39.39%	17.39%	22.92%	38.71%				
NPV[6]	97.59%	93.617%	93.61%	96.87%				
AUROC[7]	0.834	0.618	0.664	0.811	**<0.0001**	**0.0059**	0.589	0.753

[1]computer aided diagnosis [2]subgroup in which cases of thyroid entities and US characteristics identified to be associated with CAD system misdiagnosis are excluded (see table 1). [3]malignant cases or cases requiring surgery [4]McNemar test for paired comparisons, Pearson's chi squared test for unpaired comparisons [5]positive predictive value [6]negative predictive value [7]receiver operator characteristic area under the curve
